# Current Management of Hip Fracture

**DOI:** 10.3390/medicina59010026

**Published:** 2022-12-23

**Authors:** Carsten Schoeneberg

**Affiliations:** Department of Orthopedic and Emergency Surgery, Alfried Krupp Hospital, 45276 Essen, Germany; carsten.schoeneberg@krupp-krankenhaus.de

**Keywords:** hip fracture, Geriatric Trauma, orthogeriatric co-management, proximal femur fracture, Geriatric Trauma registry

This Special Issue, entitled “Current Management of Hip Fracture”, ran in the *Medicina* journal of MDPI’s “Surgery” section, reports the findings of international studies regarding different aspects in the treatment of patients suffering a proximal femur fracture. Therefore, the results of these studies are not only tailored to surgical strategies and the choice of implant, but also focus on the whole process of treating hip fracture patients. This Special Issue presents the entire treatment process. Starting with a pre-surgery risk stratification, we highlight the results of studies into different newer implants and strategies for the surgical treatment of hip fractures, as well as the impact of patient-associated factors, like malnutrition and anticoagulation, on outcomes after hip fractures are diagnosed. Two studies focus on the prevention of secondary fractures and the often-underlying osteoporosis. This issue also includes a biomechanical study which presents the impact of malposition and bone cement augmentation on fixation strength. Finally, a review of the current literature attempts to summarize the current knowledge in the treatment of hip fractures.

A total of 13 published articles demonstrate the importance of this issue and its interest to the scientific society.

Di Martino et al. present a new score, called the PRIMOF Score, to predict in-hospital mortality rates for hip fracture patients. In this retrospective study, they include over 23,000 patients, aged over 40 years, from the Abruzzo region in Italy. They split the cohort into two equal groups—the training sample and the validation sample. The final score ranges from 0 to 27 and is divided into four risk categories. This simple score, which is based on patient characteristics and clinical comorbidities, can stratify the risk of in-hospital mortality in hip fracture patients [1].

Aigner et al. analyze the effect of direct anticoagulants on the treatment of geriatric patients with a hip fracture. They conduct a registry-based analysis of 15,099 patients from the German Registry for Geriatric Trauma (ATR-DGU). They find that the time-to-surgery is prolonged in patients receiving anticoagulation drugs. However, no significant differences regarding complications, type of anesthesia and mortality are observed. They conclude that “even in the absence of widely available antidotes, the safe management of geriatric patients under DOACs with proximal femur fractures is possible” [2].

Another study from the ATR-DGU analyzes the surgical management and outcomes of pathologic hip fractures. Bliemel et al. report no differences between pathologic and osteoporotic fractures during initial hospital treatment in regard of mortality, reoperation rate and walking ability. However, in the follow-up period of 120 days, the mortality rate in pathologic hip fractures is found to be three times higher. Further, they find that pathologic per- and subtrochanteric fractures are more frequently treated by arthroplasty compared to osteoporotic fractures [3].

Pass et al. analyze the influence of malnutrition on the outcome of geriatric hip fractures, in addition to the impact of hypalbuminemia and body mass index. They conclude that “Hypoalbuminemia might be an indicator for more vulnerable patients with a compromised hemoglobin level, prothrombin time, and ASA grade. Therefore, it is also associated with higher mortality rate and postoperative complications. However, hypoalbuminemia was not an independent predictor for mortality or postoperative complications, but low albumin values were associated with a higher CCI and ASA grade than in patients with a BMI lower than 20 kg/m^2^” [4].

Giorgino et al. compare the treatment of COVID-19-positive patients in Italy and Iran suffering from proximal femur fractures in terms of characteristics, comorbidities, outcomes and complications. They find that the Italian patients are older, receive more frequent transfusions of blood during their hospital stays, and that their hospital stays are longer [5].

Niemann et al. conduct a retrospective single-center study to compare the dynamic hip screw and the femoral neck system, a recently introduced system for the internal fixation of femoral neck fractures. There is no difference in fracture complexity between both groups. They find a nearly 50% reduction in operating time and dose area product with X-Rays in the femoral neck system group. These results significantly differ, meaning that the surgical treatment using the femoral neck system results in a shorter operating time and less fluoroscopy time [6].

Hackl et al., in a retrospective single-center study, analyze the differences between valgus and anatomical reposition in Garden type III femoral neck fractures, treated with a sliding hip screw and anti-rotation screw. They exclusively include patients younger than 70 years of age. They report a significantly lower failure rate and shorter healing time in the anatomical reposition group [7].

In their retrospective single-center study, Kuner et al. analyze the outcome of intracapsular proximal femur fractures treated with the Targon^®^ FN, a dynamic locking plate fixation. They include 72 cases, in which 34 patients (47.2%) have experienced one or more complications, the most common of these being a mechanical irritation of the iliotibial band. Moreover, 46 re-operations were required. They conclude that “the Targon-FN system resulted in a high rate of post-operative complication and re-operation. Statistical analysis revealed patient age, fracture displacement, time to postoperative full weight bearing were risk factors for re-operation” [8].

One study, entitled “The influence of a Modified 3rd Generation Cementation Technique and Vacuum Mixing of Bone Cement on the Bone Cement Implantation Syndrome (BCIS) in Geriatric Patients with Cemented Hemiarthroplasty for Femoral Neck Fractures”, is published by Bökeler et al. They compare 2nd and 3rd generation cementing techniques. The incidence and early mortality are found to be significantly higher in the 2nd generation cementing technique group. Therefore, the authors decide to use a 3rd generation cementing technique [9].

A biomechanical analysis from the AO Research Institute Davos is published by Pastor et al. They compare the differences between the helical blade and the screw as head elements of the Trochanteric Femoral Nail Advanced System, either in center–center position or anterior off-center position, and the effect of bone cement augmentation. They conclude that “From a biomechanical perspective, proper centre–centre implant positioning in the femoral head is of utmost importance. In cases when this is not achievable in a clinical setting, a helical blade is more forgiving in the less ideal (anterior) position when compared to a screw, the latter revealing unacceptable low resistance to femoral head rotation and early failure. Cement augmentation of both off-centre implanted helical blade and screw head elements increases their resistance against failure; however, this effect might be redundant for helical blades and is highly unpredictable for screws” [10].

Chen et al. report the effectiveness of a fracture liaison service after 1 year of implementation at a Taipei Municipal hospital. The implementation of a fracture liaison service increases the osteoporotic treatment after hip fracture from 22.8% to 72.3% and decreases the re-fracture rate from 11.8% to 4.9%. The one-year mortality rate decreases from 17.9% to 11.8%. However, this does not reach statistical significance. They conclude that a fracture liaison service has the potential to improve the outcomes and care quality after hip fracture surgery [11].

Kraus et al. compare the awareness for osteoporosis in hip fracture patients to elderly patients undergoing elective hip arthroplasty. Although the FRAX^®^ Score is significantly different between these two groups, they determine that the fracture group has a considerably greater risk for another osteoporotic fracture and that the patients of this group show a reduced awareness of osteoporosis. Moreover, the willingness to participate in other screening programs, like colonoscopy and check-ups, is higher in both groups. The authors discuss that the reduced awareness of osteoporosis might be one factor in the low rate of osteoporotic treatment in elderly patients. They conclude that the “implementation of a screening and care program for osteoporosis such as Fracture Liaison Services (FLS) may improve patient awareness of this condition, especially among fracture patients” [12].

In a review entitled “Proximal Femoral Fractures in the Elderly: A Few Things to Know, and Some to Forget”, Maffulli and Aicale present the current literature in relation to the peri-operative, the operative, and the postoperative treatment. They define the management of hip fracture patients as a coordinated multidisciplinary approach, with early surgery, pain treatment, balanced fluid therapy, and prevention of delirium as fundamental in the treatment. The operative treatments for inter- or subtrochanteric fractures are intramedullary nailing or dynamic hip screw, and in case of neck fractures, total hip replacement or hemiarthroplasty. Early mobilization and a geriatric multidisciplinary care could be beneficial for patients with hip fracture. Because of the multifactorial reasons for hip fractures and demanding challenges, the authors concluded, that the “Management cannot be limited only to the operating theatre. Given the increase in the burden of disease, the true challenge is in prevention and in developing strategies to improve the quality of life for this group of patients” [13].

In summary, this Special Issue presents a number of studies covering the whole treatment process of hip fracture patients. A novel pre-operative risk score to estimate in-hospital mortality is presented. Several studies analyzed the outcome of modern implants and intra-operative modifications to reach a good surgical care of hip fractures. The coherence and possible optimization of post-operative osteoporotic care are presented. Finally, a review of the current literature presents the latest standards of peri-operative, operative, and postoperative treatment. Therefore, this Special Issue presents a detailed overview of the “Current Management of Hip Fracture”.

## Data Availability

Not applicable.
